# The *Drosophila *Perlecan gene *trol *regulates multiple signaling pathways in different developmental contexts

**DOI:** 10.1186/1471-213X-7-121

**Published:** 2007-11-02

**Authors:** Jonathan R Lindner, Paul R Hillman, Andrea L Barrett, Megan C Jackson, Trinity L Perry, Youngji Park, Sumana Datta

**Affiliations:** 1 Department of Biochemistry & Biophysics, Texas A&M University, College Station, Texas, 77843-2128, USA; 2 Department of Biology, Texas A&M University, College Station, Texas, 77843-2128, USA; 3 Department of Small Animal Medicine and Surgery, College of Veterinary Medicine, Texas A&M University, College Station, Texas, 77843-4474, USA; 4 Department of Cell Biology and Molecular Genetics, University of Maryland, College Park, Maryland, 20742, USA; 5 Division of Hematology and Oncology, 10900 Euclid Ave, WRB 2-104, Case Western Reserve University School of Medicine, Cleveland, Ohio, 44106, USA

## Abstract

**Background:**

Heparan sulfate proteoglycans modulate signaling by a variety of growth factors. The mammalian proteoglycan Perlecan binds and regulates signaling by Sonic Hedgehog, Fibroblast Growth Factors (FGFs), Vascular Endothelial Growth Factor (VEGF) and Platelet Derived Growth Factor (PDGF), among others, in contexts ranging from angiogenesis and cardiovascular development to cancer progression. The *Drosophila *Perlecan homolog *trol *has been shown to regulate the activity of Hedgehog and Branchless (an FGF homolog) to control the onset of stem cell proliferation in the developing brain during first instar. Here we extend analysis of *trol *mutant phenotypes to show that *trol *is required for a variety of developmental events and modulates signaling by multiple growth factors in different situations.

**Results:**

Different mutations in *trol *allow developmental progression to varying extents, suggesting that *trol *is involved in multiple cell-fate and patterning decisions. Analysis of the initiation of neuroblast proliferation at second instar demonstrated that *trol *regulates this event by modulating signaling by Hedgehog and Branchless, as it does during first instar. Trol protein is distributed over the surface of the larval brain, near the regulated neuroblasts that reside on the cortical surface. Mutations in *trol *also decrease the number of circulating plasmatocytes. This is likely to be due to decreased expression of *pointed*, the response gene for VEGF/PDGF signaling that is required for plasmatocyte proliferation. Trol is found on plasmatocytes, where it could regulate VEGF/PDGF signaling. Finally, we show that in second instar brains but not third instar brain lobes and eye discs, mutations in *trol *affect signaling by Decapentaplegic (a Transforming Growth Factor family member), Wingless (a Wnt growth factor) and Hedgehog.

**Conclusion:**

These studies extend the known functions of the *Drosophila *Perlecan homolog *trol *in both developmental and signaling contexts. These studies also highlight the fact that Trol function is not dedicated to a single molecular mechanism, but is capable of regulating different growth factor pathways depending on the cell-type and event underway.

## Background

Heparan sulfate proteoglycans (HSPGs) are a family of cell-surface and extracellular proteins modified by the attachment of glycosaminoglycan chains. The general structure of the protein core determines the family the HSPG belongs to: Syndecans contain a transmembrane domain, Glypicans are tethered to the cell surface via a GPI linkage and Perlecans are secreted components of the extracellular matrix. Both the protein core and glycan chains play important roles in HSPG function through protein-protein and sugar-protein interactions. Genetic studies, first in *Drosophila *and later in mouse and zebrafish, demonstrated the importance of the heparan sulfate chains on all three types of HSPGs for signaling by multiple growth factors such as the Fibroblast Growth Factors (FGFs), Hedgehogs, Wnts and Transforming Growth Factors (TGFβs) (reviewed in [1]).

Perlecan is the largest member of the HSPG family with a core protein of approximately 450kD in size. Perlecan has been linked to signaling by the heparan-dependent growth factors FGF2, Vascular Endothelial Growth Factor (VEGF) and Sonic Hedgehog (SHH) in mammalian systems (reviewed in [2,3]). Studies of Perlecan knock-out mice have demonstrated roles for Perlecan in vascular development and chondrogenesis as well as maintenance of basement membrane integrity [4-7]. Additional mammalian studies have revealed Perlecan's functions in angiogenesis and carcinogenesis ([8-11], reviewed in [2,12]). Mutation of Perlecan in humans leads to the muscle tone symptoms of Schwartz-Jampel syndrome, possibly through altered excitability of the neuromuscular junction and the skeletal abnormalities of Silver-Handmaker syndrome, presumably through effects on chondrogenesis [13-15].

Studies of Perlecan in invertebrate model systems have led to additional insights into Perlecan function. The single Perlecan gene in *C. elegans *is encoded by the *unc-52 *locus [16]. Mutations in *unc-52 *result in embryonic or adult paralysis due to defects in body wall muscle cells ([16,17], reviewed in [18]). Mutations in *unc-52 *also enhance cell migration defects caused by decreased netrin, FGF, TGFβ or Wnt signaling. In *Drosophila*, Perlecan is encoded by the *trol *gene on the X chromosome [19,20], which was initially implicated in the control of stem cell division in the developing larval brain [21,22]. In the larval brain, *trol *promotes the cell cycle progression of mitotically arrested neuroblasts [23,24] through modulation of FGF and Hedgehog signaling [19]. These *Drosophila *studies were the first to link Perlecan to Hedgehog signaling. More recently, studies of oogenesis in *Drosophila *have uncovered a role for Perlecan in the maintenance of epithelial cell polarity through interactions with the extracellular matrix receptor Dystroglycan [25].

The many signaling pathways associated with HSPGs in general and Perlecan in particular led us to ask what other biological processes may require Perlecan function. We used a series of *trol *mutants to investigate several phenotypes ranging from overall developmental progress to specific alterations of stem cell division and hemocyte production. Furthermore, analysis of signaling pathway response genes revealed that while mutations in Perlecan decrease signaling in multiple pathways, at least some of these effects are tissue specific.

## Results and discussion

### Development and lethal phase

**Figure 1:**
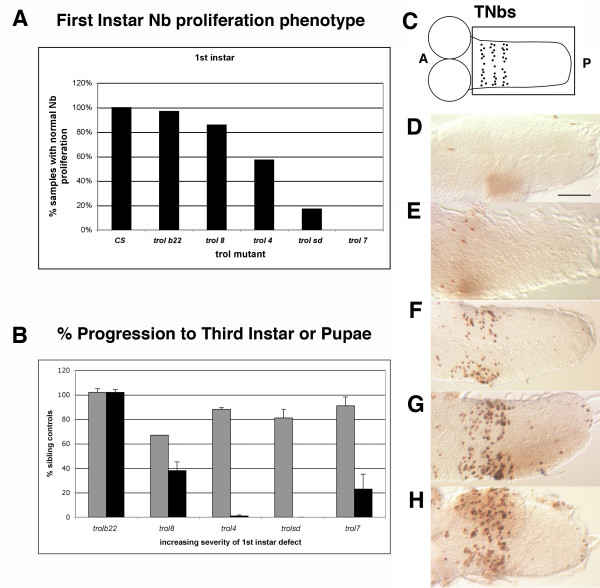
**Phenotypic series of *trol *alleles**. A) First instar neuroblast proliferation phenotype presented as % of samples with numbers of BrdU labeled neuroblasts falling within the control range, some data originally published in Park et al, 2003a. B) Lethal phase phenotype presented as the percentage of *trol *mutant animals capable of survival and development to third instar (grey bars) or to pupal formation (black bars) compared to sibling controls. Error bars indicate s.e.m. C) Cartoon of second instar larval brain with dividing TNBs in ventral ganglion. Boxed area indicates portion of brain shown in panels D-G below. A = anterior, P = posterior. D-G) Examples of the five classes of BrdU incorporation into TNbs are shown. In all panels anterior is to the left, posterior is to the right. Scale bar in panel D indicates 25 um. D) None (class 1). E) Few (class 2). F) Segmentally repeated lines with few extra neuroblasts (class 3). G) Segmentally repeated lines with several scattered neuroblasts (class 4). H) Heavily populated segmental pattern (class 5).

We had previously shown that the viable *trol*^*b*22 ^and the lethal *trol*^8^, *trol*^4^, and *trol*^*sd *^alleles form an allelic series of increasing severity based on their onset of neuroblast proliferation phenotype in first instar larval brain lobes [24]. Identification [19] and phenotypic analysis of a fifth *trol *allele, *trol*^7^, revealed that *trol*^7 ^is the strongest allele with respect to the first instar proliferation phenotype (Fig. 1A). Unexpectedly, *trol*^7 ^mutant larvae appeared healthier overall than other *trol *mutant larvae, suggesting that the order of allelic severity determined by analysis of first instar brain lobes would be different from one based on developmental progression. To test this hypothesis, we examined the lethal stage and developmental progression of larvae mutant for *trol*^*b*22^, *trol*^8^, *trol*^4^, *trol*^*sd *^and *trol*^7^. In all the experiments, crosses were designed to use sibling controls in order to minimize the effects of genetic background, which can be significant in fly stocks kept in reproductive isolation from each other for years in our laboratory. For the lethal *trol *alleles, *y trol*^*x *^*/Binsn *stocks were used as the source of mutant and control larvae. At this stage of first instar, mutations in *y *produce one of the few reliable phenotypic markers. Thus *trol *mutant animals were identified as *y *mutant larvae that are *y trol*^*x *^hemizygous males and sibling controls were a mixed population of *y*^+ ^animals: *y trol*^*x*^*/Binsn *heterozygotes, *Binsn *homozygous females and *Binsn *hemizygous males. Note that while *Binsn *homozygous females and hemizygous males can become viable adults, not all *Binsn/Binsn *or *Binsn/Y *larvae reach adulthood. Thus our comparison provides a measure of developmental progression and lethal phase that will err on the side of minimizing the *trol *mutant phenotype. For analysis of the viable *trol*^*b*22 ^allele, additional crosses were required to produce wild-type sibling controls from the homozygous *y trol*^*b*22 ^stock. *y trol*^*b*22 ^animals were crossed to the wild-type strain Canton Special (CS) to produce *trol*^*b*22^/CS heterozygous females. These females were mated to CS males to generate hemizygous *y trol*^*b*22 ^male larvae and *y*^+ ^sibling control larvae (a mixture of heterozygous *y trol*^*b*22^*/CS *female, homozygous *CS *female and hemizygous *CS *male) for the developmental studies. One hundred mutant and sibling control animals for each allele were collected at early first instar and monitored at 24 hour intervals for developmental progression and viability. Of these, only 1 mutant *trol*^4 ^and no *trol*^*sd *^animals pupariated. However, when the same numbers of *trol*^*b*22^, *trol*^8 ^and *trol*^7 ^mutant larvae were analyzed and compared to sibling controls, 102%, 38% and 23% of the animals were able to pupariate, respectively (Fig. 1B). The pupariation assay resulted in shifts of perceived functional severity for both *trol*^4 ^and *trol*^7^, with *trol*^4 ^appearing stronger and *trol*^7 ^appearing weaker.

Why would animals mutant for *trol*^7 ^(that has a strong effect on neuroblast proliferation) be able to progress further in development than animals mutant for *trol*^4 ^which causes a weaker neuroblast proliferation phenotype? One possibility is that *trol *modulates the activity of different signaling pathways in different tissues. For example, a mutation that affects the ability of Trol to function in the Hh pathway would have a severe effect on developmental decisions that require Hh activity and very little effect on decisions that do not require Hh signaling. To address this possibility we investigated the impact of *trol *mutations on two distinct developmental events and several signaling pathways.

### Effects of trol mutations on TNb proliferation

*trol *was initially identified as a mutation on the X chromosome that affected the proliferation pattern of neuroblasts in the brain lobes and ventral ganglion [21,22]. Since neuroblasts in the thoracic region of the ventral ganglion begin proliferation in early second instar [21,26,27], we evaluated the ability of thoracic neuroblasts (TNbs) to enter S phase in *trol *mutant animals. We adapted the idea of phenotypic classes to produce a scale for the extent of TNb proliferation at four hours post molt (Fig. 1C–G). Five TNb classes were defined as follows: Class 1, no neuroblasts labeled; Class 2, a small number of labeled neuroblasts with no distinct segmental pattern; Class 3, labeled neuroblasts in a segmentally repeated lines with very few labeled neuroblasts in between the lines or in the medial region of the ventral ganglion; Class 4, labeled neuroblasts in a segmentally repeated line with some labeled neuroblasts in between the lines or in the medial region of the ventral ganglion; and Class 5, labeled neuroblasts in heavily populated segmental pattern with many labeled neuroblasts in the medial portion of the ventral ganglion. When both sides of a ventral ganglion did not conform to a single class, the sample was scored as the higher class. This will have a conservative effect of scoring a partial loss-of-proliferation TNb phenotype as more wild-type. Thus we can have greater confidence in the significance of TNb proliferation phenotypes observed compared to controls.

We first examined the onset of TNb proliferation in wild-type sibling controls to determine the time point at which to assay the *trol *mutants (data not shown). In our hands, high levels of 5-Bromodeoxyuridine (BrdU)-labeled TNbs were first observed in control samples between 2–5 hours post molt depending on genetic background. This timing is slightly earlier than the previous observation that TNb mitosis begins between 28–34 hours post hatching, or 4–10 hours post molt (pm) to second instar [26]. To evaluate the TNb proliferation phenotype produced by the different *trol *alleles, at least twenty samples for each mutant and sibling control (generated as described above) were allowed to incorporate BrdU from 4–5 hours pm and scored for TNb class. The average score and standard error of the mean were calculated for each group of sibling controls. The control value for each study was set to a value of 4 to control for genetic background effects between experiments. Setting controls to a value of 4 on our 5 point scale was chosen to allow evaluation of over-proliferation (>4) as well as under-proliferation (<4) mutant phenotypes. To obtain the TNb phenotype score for each mutant allele we normalized the score for each sample to the respective sibling control and calculated the average and standard error of the mean (Table 1). Surprisingly, *trol*^*b*22 ^mutants had a significantly higher than normal level of TNb proliferation (TNb score >4) while the remaining *trol *mutants showed decreased TNb cell division compared to controls. The differences between mutant and control BrdU incorporation were statistically significant (p < 0.05) for each mutant allele. Comparison between mutants showed a phenotypic trend from *trol*^*b*22 ^having hyperactive TNb proliferation to *trol*^*sd *^as the mutant with the fewest labeled TNbs. In this assay *trol*^7 ^mutants appear to have a weaker phenotype than *trol*^*sd*^.

**Table 1:** TNb BrdU incorporation phenotype of *trol *mutants at 4–5 hours pm.

***trol *allele**	TNb score*	**S.E.M.**
Control	4.00	0.27
*trol*^*b*22^	4.55	0.16
*trol*^8^	3.37	0.21
*trol*^4^	3.00	0.27
*trol*^7^	2.95	0.2
*trol*^*sd*^	2.45	0.21

* All scores significantly different from controls at p < 0.05.

### trol affects Bnl and Hh signaling in the ventral ganglia

**Figure 2:**
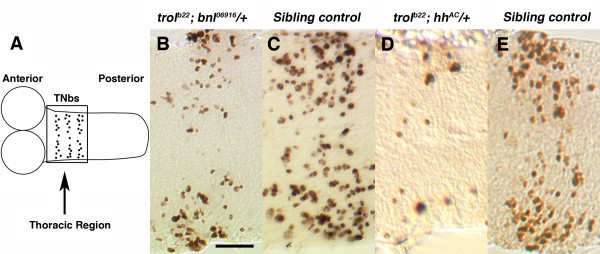
**Trol modulates Hh and Bnl signaling in the ventral ganglion**. A) Cartoon of second instar brain indicating location of TNbs. Boxed area outlines thoracic region shown in panels B-E. One hour BrdU incorporation in TNbs in B) a *y trol*^*b*22^; *bnl*^06916^/+ brain from 2–3 hours pm; C) a sibling control *y trol*^*b*22^*/+ ; *+/*TM3y*^+^, *y trol*^*b*22 ^; +/*TM3y*^+ ^or *y trol*^*b*22^/+ ; *bnl*^06916^*/+ *brain 2–3 hours pm (see text); D) a *y trol*^*b*22^; *hh*^*AC*^/+ brain from 2–3 hours pm and a E) sibling control *y trol*^*b*22^*/+ ; *+/*TM3y*^+^, *y trol*^*b*22 ^; +/*TM3y*^+ ^or *y trol*^*b*22^/+ ; *hh*^*AC *^*/+ *brain from 2–3 hours pm (see text). Scale bar in panel A indicates 10 um.

To determine if *trol *affects TNb proliferation through modulation of Bnl and Hh signaling, we used genetic interaction studies with the weak *trol *allele *trol*^*b*22^. As we have shown, *trol*^*b*22 ^animals have over proliferation of TNbs compared to sibling controls (Table 1). For the genetic interaction assay, *y trol*^*b*22 ^females were crossed to *bnl*^06916^*/TM3y*^+ ^males to generate *y *larvae that were y *trol*^*b*22 ^; *bnl*^06916^/+ and *y*^+ ^sibling controls that were a combination of *y trol*^*b*22 ^*; +/TM3y*^+ ^males, *y trol*^*b*22^/+ *; +/TM3y*^+ ^females and *trol*^*b*22^/+ *; bnl*^06916^*/+ *females. None of the sibling controls had TNb proliferation scores outside of the normal (CS) range at this timepoint. *y trol*^*b*22 ^males carrying a single copy of the *bnl*^06916 ^allele had fewer BrdU labeled TNbs at 2–3 hours post molt to second instar (pm) compared to siblings that were hemizygous or heterozygous for *trol*^*b*22 ^alone or heterozygous for both *trol*^*b*22 ^and *bnl*^06916 ^(Fig. 2). The decreased TNb proliferation in samples heterozygous for *bnl*^06916 ^in a *trol*^*b*22 ^background compared to controls versus the increased proliferation in *trol*^*b*22 ^animals wild-type for *bnl *compared to controls suggests that the *trol*^*b*22 ^mutation affects signaling by Bnl in the ventral ganglion at second instar.

**Figure 3:**
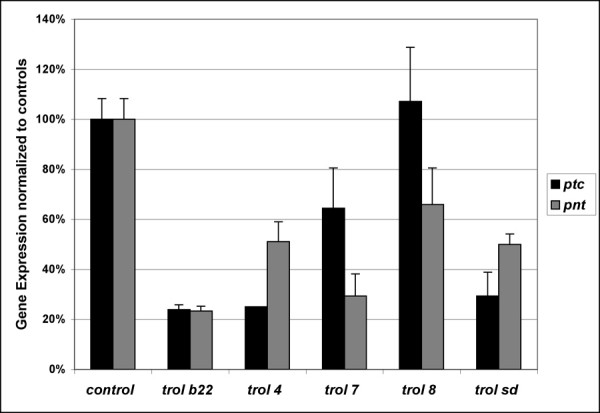
**Hh and Bnl signaling activity in the ventral ganglion of *trol *mutant animals**. Quantitative RT-PCR analysis of the expression of the Hh response gene *ptc *(black bars) and the Bnl response gene *pnt *(grey bars) in the ventral ganglia of *trol *mutant normalized to controls at one hour post molt to second instar. β*-actin *was used to as an internal control to normalize message levels. All analyses were done in triplicate and three different concentrations to ensure samples were within linear range of amplification. Error bars indicate standard deviation.

We also used genetic interactions to evaluate the possibility that *trol *might affect Hedgehog signaling in the ventral ganglion. For this study *y trol*^*b*22 ^females were crossed to *hh*^*AC *^*/TM3y*^+ ^males to generate *y *larvae that were y *trol*^*b*22 ^; *hh*^*AC*^/+ and *y*^+ ^sibling controls that were a combination of *y trol*^*b*22 ^*; +/TM3y*^+ ^males, *y trol*^*b*22^/+ *; +/TM3y*^+ ^females and *trol*^*b*22^/+ *; hh*^*AC *^*/+ *females. *trol*^*b*22 ^animals carried a single copy of the *hh*^*AC *^allele, also had fewer dividing TNbs at 2–3 hours pm compared to sibling controls (Fig. 2). The decrease in the number of BrdU labeled TNbs in *trol*^*b*22 ^hemizygotes upon heterozygosity for *hh*^*AC *^suggest that mutations in *trol *also weaken the signaling action of Hh in the ventral ganglion. To further test our hypotheses, we examined the signaling activity of Bnl and Hh in *trol *mutants directly by quantitative RealTime PCR (qRT-PCR) in the central nervous system (CNS). To avoid interfering signals from the lobes of the second instar brain that might overwhelm differences in signal in the ventral ganglion, we isolated ventral ganglia from second instar *trol *mutant and sibling control brains at one hour post molt. First instar brains were dissected at 20 hours post hatching which correlates with the end of the BrdU labeling period used to assess neuroblast proliferation in first instar ([24] and this manuscript). RNA was isolated, cDNA synthesized and amplified and the level of expression of the Hh response gene *ptc *(Fig. 3) and the Bnl response gene *pnt *(Fig. 3) assayed. Our qRT-PCR data demonstrate that mutations in *trol *affect the strength of signaling by both Hh and Bnl in the larval ventral ganglion and in first instar larval brains (data not shown).

### Trol localization in the larval brain

**Figure 4:**
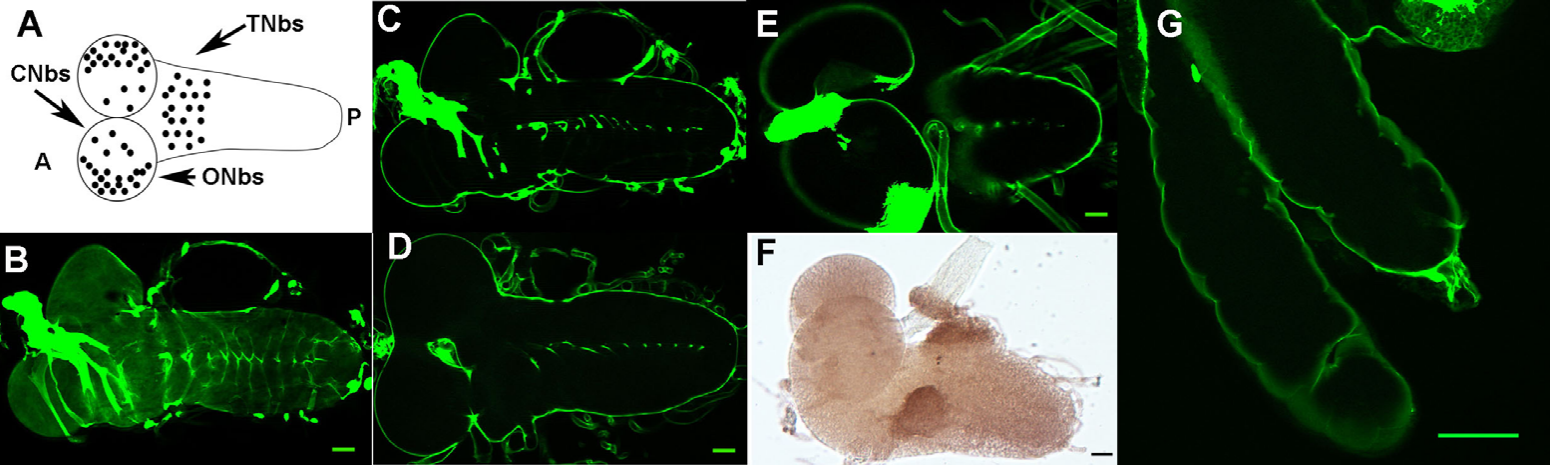
**Localization of Trol-GFP in the larval brain**. A) Schematic of neuroblast position in the larval brain. A = anterior, P = posterior, ONbs = optic lobe neuroblasts, CNbs = central brain neuroblasts, TNbs = thoracic neuroblasts. In panels B-F, Anterior is to the left, Posterior is to the right. B) Optical section of Trol-GFP brains at first instar, brain surface, scale bar indicates 10 um for both panels B and C. C) Trol-GFP localization in first instar internal section. D) Trol-GFP localization in second instar internal section, scale bar indicates 15 um. E) Trol-GFP localization in third instar internal section, scale bar indicates 25 um. F) First instar brain stained with anti-Trol antibody, showing staining over the entire surface of the brain. Scale bar indicates 10 um. G) Trol-GFP localization in internal section of third instar salivary gland. Scale bar indicates 25 um.

Previously we had isolated complexes containing either Trol and FGF2 or Trol and Hh by co-immunoprecipitation [19]. In combination with our genetic studies, these complexes suggested that the Trol protein regulates neuroblast division by binding growth factors that stimulate neuroblast proliferation in a manner similar to Perlecan-mediated promotion of ligand-receptor binding described in mammalian systems [28]. This model predicts that Trol protein should be localized near the regulated neuroblasts, i.e. the optic lobe and central brain neuroblasts of the first instar brain [19-24,29] and the thoracic region of the second instar brain ([22] and Fig. 4A). In contrast, *in situ *hybridization studies in the third instar larval brain by Voigt et al [20] had revealed that only a few isolated cells at a distance from the optic lobe proliferation centers express *trol*. This led the authors to suggest that Trol is unlikely to regulate neuroblast proliferation by promoting binding of FGF-type ligands to their receptors since this would require Trol protein localization near the responding cells. However, since Trol is a secreted protein with a long half-life, mRNA expression patterns may not accurately portray protein localization. In addition, Voigt et al conducted their *in situ *analysis at late third instar, 2–3 days after the activation of neuroblast division at late first or early second instar. Thus the expression pattern observed for *trol *message at late third instar may not reflect expression of *trol *at earlier larval stages. Furthermore, a study of *trol *mRNA localization by *in situ *hybridization in embryos showed either no obvious staining in the CNS [30] or expression in a small subset of glial cells in the CNS [20]. However, analysis of Trol protein localization with an anti-Trol antibody in embryos revealed localization to the basement membrane of the CNS [30]. This evidence further suggests that *trol *message patterns may not reflect Trol protein localization. To address the conflicting models, we took advantage of a Trol protein trap in which the GFP gene is inserted within the endogenous *trol *locus [31]. Analysis of GFP localization in larval brains demonstrates that Trol-GFP is found in a layer, presumably the basal lamina, encompassing the entire outer surface of the larval brain with little to no signal detectable at internal sites within the brain (Fig. 4B–E). Trol-GFP was also observed in the basal lamina surrounding nerves emanating from the larval brain. The distribution of Trol over the entire brain was further verified by immunohistochemistry using an anti-Trol antibody (Fig. 4F). This localization of the Trol protein is consistent with the model that Trol binds Bnl and Hh and facilitates their signaling to promote neuroblast proliferation, as the regulated neuroblasts are found at the surface of the cellular cortex in both the brain lobes and the ventral ganglion [21,26,32]. To determine if the localization of Trol-GFP to the basal lamina was unique to the larval brain, we examined Trol-GFP in the salivary glands. As in our larval brain studies, Trol-GFP is found on the surface of the gland, presumably as a component of the basal lamina (Fig. 4G).

### Effects of trol mutations on larval hemocyte number

**Figure 5:**
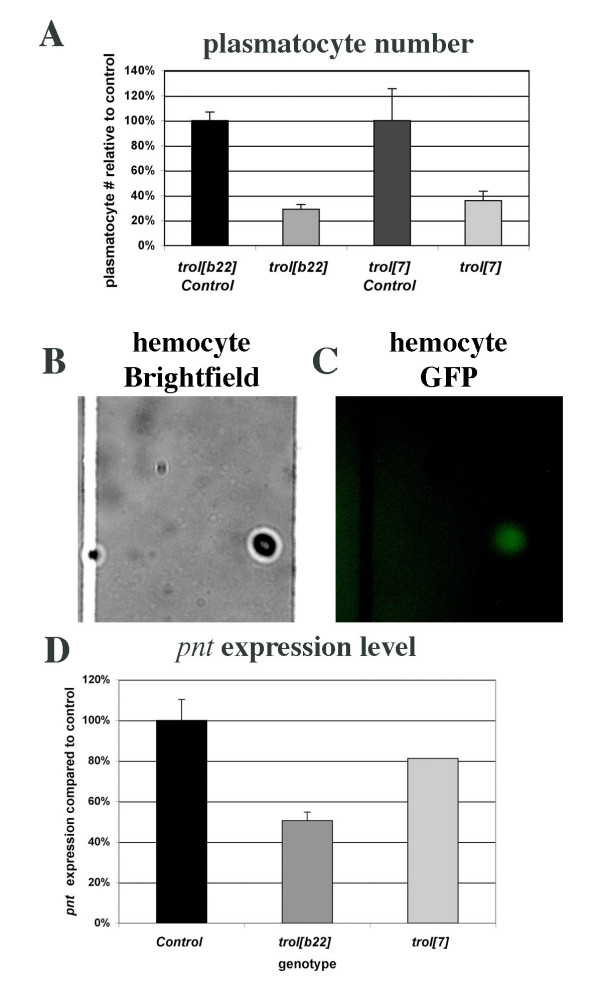
**Mutations in *trol *decrease circulating plasmatocyte number and *pnt *expression**. A) Quantification of circulating plasmatocytes in *trol*^*b*22 ^and *trol*^7 ^mutants compared to controls. Each sample consisted of hemolymph pooled from three third instar larvae. Five squares were counted for each sample. Each genotype was analyzed in triplicate. B) Brightfield image of plasmatocytes from Trol-GFP stock. C) Fluorescence image of plasmatocytes from Trol-GFP stock demonstrating presence of Trol on plasmatocytes. D) Expression of the VEGF/PDGF response gene *pnt *in *trol*^*b*22 ^and *trol*^7 ^mutant hemocytes compared to sibling controls by qRT-PCR. Samples of hemolymph from three third instar larvae of each genotype were pooled, RNA extracted, amplified and analyzed. All reactions were carried out in triplicate at three different template concentrations to ensure amplification was in the linear range. β*-actin *was used as an internal normalization control.

A second system where we thought *trol *might have an effect on development is the production of hemocytes during larval life. A number of studies have elegantly shown that the larval lymph gland is the source of larval hemocytes [33]. In the primary lobe of the third instar lymph gland prohemocytes arise in the medullary zone while maturing hemocytes are found in the adjacent cortical zone. Hemocytes are then released into the hemolymph and are present as three types of circulating cells: plasmatocytes (95%), lamellocytes (1–5%) and crystal cells (rare). Each cell-type has characteristic morphology and can be easily identified under a compound microscope. Mature circulating larval hemocytes are still undergoing cell division, albeit at a low rate, as shown by staining of hemocytes with phosphohistone H3, an M phase marker [34,35]. Expression of an activated Ras (Ras^v12^) in circulating hemocytes increases the percentage of circulating hemocytes that stain for phosphohistone H3 and results in a 40-fold increase in the number of hemocytes through activation of the Ras-MAPK pathway [34]. The Ras-MAPK pathway is activated by Vascular Endothelial Growth Factor (VEGF) and Platelet Derived Growth Factor (PDGF) among others. Signaling by mammalian homologs of both growth factors has been linked to mammalian Perlecan [3]. Furthermore, studies of PDGF/VEGF receptor (*PVR*) in *Drosophila *revealed that *PVR *is expressed in plasmatocytes and that decreased PVR function leads to increased hemocyte cell death [33]. Thus it seemed likely that mutations in *trol *could decrease PDGF/VEGF signaling in circulating plasmatocytes, resulting in decreased numbers of circulating plasmatocytes in *trol *mutants. To address this hypothesis, we determined the relative number of circulating plasmatocytes in third instar *trol*^*b*22 ^or *trol*^7 ^and sibling control larvae (Fig. 5A). Our analysis demonstrates a significant (p < 0.05) drop in the number of plasmatocytes in *trol *mutant versus sibling control larvae.

### Trol localization and function in hemocytes

The decrease in the number of circulating plasmatocytes in *trol *mutants versus controls suggested that *trol *might indeed function to promote Ras-MAPK signaling by PDGF/VEGF in circulating plasmatocytes. This predicts that Trol protein would be localized on these plasmatocytes. We used Trol-GFP protein trap to examine the plasmatocytes for the presence or absence of Trol protein. Fluorescence microscopy revealed that Trol-GFP is indeed found on circulating plasmatocytes in third instar larvae (Fig. 5B,C), but not in the lymph gland (data not shown).

This result is consistent with the requirement for Ras-MAPK activation in plasmatocytes for plasmatocyte proliferation and for PVR in plasmatocytes to avert apoptosis, and supports the hypothesis that Trol modulates PVR-Ras-MAPK signaling in plasmatocytes. The ETS-transcription factor *pnt *is a MAPK-response gene and will drive plasmatocyte proliferation [36]. Therefore we asked if *trol *mutant plasmatocytes show decreased levels of *pnt *compared to controls. Plasmatocytes were collected by bleeding third instar *trol*^*b*22 ^and *trol*^7 ^mutant larvae and sibling controls, RNA was extracted and amplified, and subjected to qRT-PCR analysis. qRT-PCR studies demonstrated that plasmatocytes isolated from either *trol*^*b*22 ^or *trol*^7 ^mutants show decreased expression of *pnt *compared to controls, further evidence that *trol *modulates Ras-MAPK signaling in plasmatocytes (Fig. 5D).

### Trol and other growth factor signaling pathways

**Figure 6:**
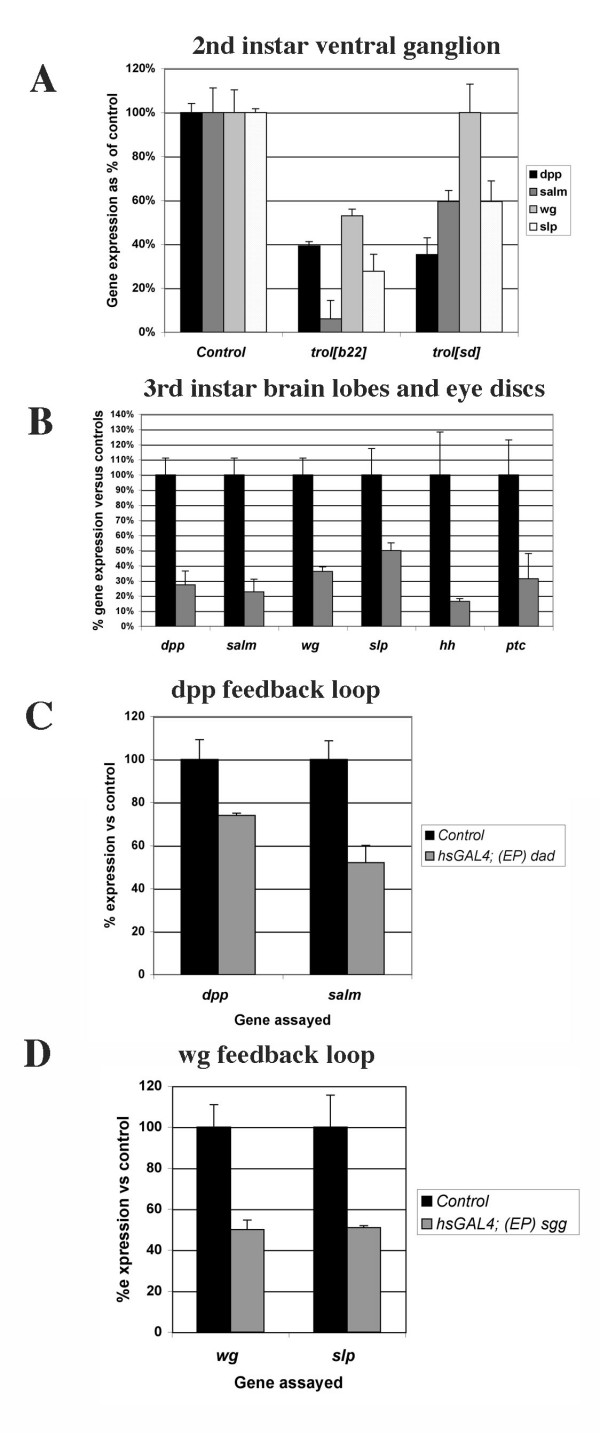
**Dpp, Wg and Hh signaling are affected in *trol *mutants in second instar brains but not in third instar brain lobes/eye discs**. qRT-PCR analysis of A) the expression levels of *dpp*, its response gene *salm*, *wg *and its response gene *slp *in the ventral ganglia of second instar larvae. Data for the Hh response gene *ptc *is shown in Fig. 3. B) Expression levels of *dpp*, its response gene *salm*, *wg *and its response gene *slp*, *hh *and its response gene *ptc *in *trol*^*sd *^mutant third instar brain lobes/eye discs. C) Expression of *dpp *and *salm *in *hsGAL4/+; +/(EP)dad *second instar ventral ganglia and *hsGAL4 *controls. D) Expression of *wg *and *slp *in *hsGAL4/+; +/(EP)sgg *second instar ventral ganglia and *hsGAL4 *controls. In all panels, error bars indicate standard error. All reactions were carried out in triplicate at three different template concentrations to ensure amplification was in the linear range. β*-actin *was used as an internal normalization control.

Two other growth factor signaling pathways that have been linked to HSPGs are the *wingless *(*wg*/Wnt) and *decapentaplegic *(*dpp*/TGFβ) signaling pathways. Both of these pathways are active in the developing *Drosophila *eye disc and/or third instar brain along with Hh and Ras-MAPK signaling [37,38]. To ask if Trol might modulate the Dpp and Wg pathways we evaluated the expression of *dpp *and *wg *and their target genes *spalt major *(*salm*, [39]) and *sloppy paired *(*slp*, [40]), respectively, in second instar ventral ganglia and third instar brains and eye discs from *trol *mutant larvae by qRT-PCR (Fig. 6A–B). We also assayed expression of *hh *and its response gene *ptc *in third instar brains and eye discs. In the *trol*^*b*22 ^second instar ventral ganglion we observed a significant drop in the level of both *dpp *and *wg *compared to controls. The *trol*^*b*22 ^mutation also resulted in diminished signaling efficiency by both growth factors as indicated by a larger drop in the level of their response genes *salm *and *slp *compared to the ligands themselves. In contrast, the *trol*^*sd *^mutation decreased only *dpp *expression, but the efficiency of both *dpp *and *wg *signaling was impaired. Thus in the second instar ventral gangion, wild-type function of *trol *appears to be required for normal signaling by *hh*, *bnl*, *dpp *and *wg *(Figs. 3 and 6A). The decreased expression of *dpp *and *wg *in *trol*^*b*22 ^mutants and of *dpp *in *trol*^*sd *^mutants may be due to secondary effects on *dpp *and *wg *expression caused by the changes in Hh and Bnl signaling in trol mutants. Alternatively, decreased expression of *dpp *and *wg *could be due to positive feedback between Dpp signaling and *dpp *expression and Wg signaling and *wg *expression, respectively. To test the latter possibility, we blocked Dpp signaling by over-expression of *daughters against dpp *(*dad) *[41], and assayed for *dpp *message levels (Fig. 6C). *(EP)dad *females were crossed to *hsGAL4 *males to drive expression of *dad*. Embryogenesis and first instar larval development were carried out at 18°C to limit expression of *dad *and inhibition of Dpp signaling at early stages. Upon molt to second instar, larvae were moved to 25°C for one hour to induce expression of *dad*. Larval brains were dissected and the ventral ganglia harvested for RNA isolations. Inhibition of Dpp signaling was confirmed by analysis of *salm *mRNA levels. Similarly, we inhibited Wg signaling by over-expression of *shaggy *(*sgg*), and assayed for *wg *message levels (Fig. 6D). Decreased Wg signaling was verified by analysis of *slp *expression levels. As shown by our qRT-PCR analysis, inhibition of Dpp signaling by over-expression of *dad *resulted in a drop in expression of the *dpp *ligand itself. Inhibition of Wg signaling by over-expression of *sgg *also produced a drop in the expression of *wg*. As these studies were conducted in flies wild-type for *trol*, they eliminate the possibility that the decreased expression of *dpp *and *wg *in *trol *mutants was due solely to reduced Trol-mediated signaling by Hh and/or Bnl. These data indicate the presence of a positive feedback loop for Dpp and Wg in the ventral ganglion.

To determine if Trol is necessary for growth factor signaling in other tissues at other stages we assayed for *dpp*, *wg *and *hh *expression and activity in *trol*^*b*22 ^and *trol*^*sd *^third instar brain lobes and eye discs. No significant changes in either growth factor expression or signaling were observed in *trol*^*b*22 ^samples (data not shown). In *trol*^*sd *^samples, expression of all three growth factors decreased by 65–85%, as did the expression of their response genes (Fig. 6B). The sole exception is *wg/slp*, where *wg *expression decreased about 65% and *slp *expression decreased only about 50%. These data indicate that mutations in *trol *do not dramatically decrease the signaling efficiency of Dpp, Wg or Hh in third instar brain lobes and eye discs, unlike the effect of those same *trol *mutations in second instar.

## Conclusion

### trol and Drosophila development

We have previously demonstrated that mutations in *trol *prevent the onset of neuroblast division in the first instar brain and that most *trol *mutations are lethal. Mutations in a second gene, *anachronism*, also affect the onset of neuroblast proliferation but in the opposite manner: in *anachronism *mutants, mitotically regulated neuroblasts begin cell division too early [42]. However, when a lethal *trol *mutation was combined with a viable allele of *anachronism*, the lack of neuroblast division was rescued (double mutants exhibited the *anachronism *phenotype of premature neuroblast division) but lethality was not [21]. This outcome suggested that *trol *function is required for other developmental events necessary for survival. Further analyses revealed that *trol *modulates Hh and Bnl signaling in the first instar brain [19]. Here we have demonstrated that *trol *function is required for developmental progression to third instar and for pupariation. Analogous to its function in the first instar brain, *trol *is required to initiate the division of a second, independent and spatially distinct population of neuroblasts in the second instar brain (Table 1, Fig. 1). This initiation of division is also dependent on Bnl and Hh signaling (Fig. 2). We have also demonstrated that the Trol protein is localized to the surface of the brain at all larval stages, which places it in close proximity to the regulated neuroblasts. This localization is consistent with our model where Trol regulates Bnl and Hh signaling to cells adjacent to the regulated neuroblasts by binding the growth factors directly [19]. Trol protein localization to the basal lamina is not limited to the larval brain, as Trol-GFP studies also showed Trol protein in the basal lamina surrounding the salivary glands (Fig. 4G). *trol *function is not limited to the nervous system, as mutations in *trol *also diminish the number of circulating plasmatocytes by decreasing expression of *pnt*, a PVR response gene in plasmatocytes (Fig. 5). We speculate that *trol *may be necessary for signaling by the *Drosophila *PDGF and/or VEGF growth factor, just as mammalian Perlecan has been shown to function during angiogenesis [3]. Our studies of Dpp and Wg indicate a positive feedback between *dpp *expression and Dpp signaling and *wg *expression and Wg signaling in the second instar ventral ganglion. Signaling by Dpp and Wg is also dependent on *trol *in the second instar brain, but not (or very little) in the third instar brain lobes and eye discs (Fig. 6), despite the fact that Dpp and Wg signaling are taking place in those tissues. In fact, even Hh signaling appears to be independent of *trol *in this context. These results highlight an important concept in *trol*, and indeed, in proteoglycan function: that the Trol protein will be used at different times and places to regulate the signaling of different growth factors. Deciphering the role of *trol *in different developmental decisions will require that we examine each event individually, as *trol *will not necessarily mediate the same molecular mechanism each time.

### Involvement of HSPGs in growth factor signaling

The requirement for heparan sulfate proteoglycans in signaling by different families of growth factors is well established [43], but what is not yet clear is why different organs and tissue types use different HSPGs to modulate these signaling pathways. One possibility is that the specific mechanism(s) through which these molecules modulate signaling activity allows for site-specific variations in the regulation of signaling activity. HSPGs with varied amino acid sequence can act in the same signaling pathway, such as Syndecan-4 and Perlecan for FGF2 [28,44] or Glypicans, Syndecan-3 and Perlecan for Hh [19,43,45]. Mutations that affect heparan sulfate synthesis or modification strongly affect FGF2 and Hh signaling [43]. Furthermore, Perlecan isolated from various endothelial cell sources has different binding affinities for FGF2 [46]. These data initially suggested that the protein core of the HSPG might have little to do with signaling specificity and that the main functional domain of HSPGs is concentrated in the sequence of the heparan sulfate chains.

The carbohydrate-centric view is being challenged by studies that indicate a role for the protein-protein interactions of HSPGs with growth factors and other signaling molecules. For example, expression of chimeric molecules has shown that the cytoplasmic tail of Syndecan is specifically required for FGF2 signaling in addition to its heparan sulfate chains [47]. Perlecan protein-protein interactions include the ability of Perlecan to bind growth factors and extracellular matrix molecules at various sites on its protein core. Further mechanisms that allow for differential regulation include processing of HSPGs. These studies suggest a reason for the use of a particular HSPG during an individual developmental decision – the flexibility of combining both carbohydrate-based regulation and protein-based regulation of cell-cell signaling may make a specific HSPG uniquely suited for a given situation.

In the context of combined carbohydrate and protein inputs into HSPG function, it becomes clear that a given HSPG may be expressed and function in very specific contexts that take advantage of its unique regulatory abilities. It is interesting to note that we have connected Perlecan with FGF and Hh signaling in the developing fly brain while mouse studies have shown that Perlecan knock-out mice have cerebral cortex abnormalities [6,19,21]. *trol *mutant larvae have decreased numbers of circulating hemocytes that are likely due to decreased Ras-MAPK signaling by VEGF/PDGF. Perlecan knock-out mice also have defects in chondrogenesis and cardiovascular development and mammalian studies have demonstrated a role for Perlecan in angiogenesis driven by FGFs, VEGF and PDGF [3]. Finally, we have shown that Perlecan is required for SHH signaling during human prostate cancer growth [8], which reveals a new system for the investigation of the mechanism of Perlecan action. Further analysis of the ability of HSPGs to substitute for each other in cell fate decisions and the means by which they individually regulate cell-cell communication will lead to a clearer understanding of the inputs necessary for cells to carry out a developmental or disease progression.

## Methods

### Fly stocks

Stocks of the viable *trol*^*b*22 ^allele and the lethal *trol*^4^, *trol*^7^, *trol*^8 ^and *trol*^*sd *^alleles have been described previously [19,21,22,29]. All *trol *mutant stocks with the exception of *trol*^*b*22 ^are *y trol*^*x*^*w/Binsn *where the chromosome carrying the *trol *mutation is marked with *y *to facilitate identification of *y trol *mutant versus *y*^+^control larvae. The *trol-GFP *protein trap was obtained from Dr. Stephane Noselli. The *bnl*^06916 ^and *hh*^*AC *^stocks were obtained from the Bloomington stock center and used to construct *y ; bnl*^06916^*/TM3y*^+ ^and *y ; hh*^*AC*^*/TM3y*^+ ^stocks for genetic studies.

### Lethal phase

Early first instar larvae were collected and placed on apple juice plates with yeast. Each plate initially had 50 mutant or control animals per plate, segregated to prevent competition between mutant and wildtype siblings. Two plates of each genotype were examined. The number and stage of larvae still present on each plate were assayed every 24 hours and the survivors transferred to a fresh plate. Since none of the *trol *mutants with the exception of *trol*^*b*22 ^produce viable adults, individual animals were followed only until pupariation.

### Developmental staging

Developmental synchronization was carried out as previously described [19,21,23,48]. Flies were allowed to lay eggs on apple juice agar plates with fresh yeast overnight or for about 24 hours. For staging of synchronized first instar larvae, the plate was first cleared of any larvae and newly hatched larvae collected in one hour windows and placed on new apple juice plates with yeast at 25°C for aging. For staging of second instar larvae, late first instar larvae were placed on fresh apple juice plates with yeast. Newly molted second instars were collected in one hour windows and placed on apple juice plates with yeast at 25°C for aging or dissected immediately.

### Proliferation assay

BrdU assays were carried out as previously described [19,21,23,48]. Briefly, animals were fed BrdU-containing artificial medium for one hour, dissected in PBST and fixed with Histochoice (Amresco) for 10 minutes. Brain samples were denatured in PBST-HCl for 30 minutes, washed and blocked in PBNT for one hour. Primary anti-BrdU antibody (Becton-Dickinson) was added at 1:200 overnight at 4°C. Samples were washed and incubated with HRP-conjugated secondary antibody at 1:400 for 2–4 hours at room temperature. Signal was developed using a DAB substrate (Sigma).

### Larval hemocyte assay

Hemocytes from three third instar larvae were harvested using a Pasteur pipette pulled to generate a capillary end, pooled and counted on a standard hemacytometer slide. Five 16-square regions were counted for each pooled sample. Three replicates were assayed for each genotype.

### Quantitative RealTime PCR

Whole first instar brains or ventral ganglia dissected from the brains of second instar larvae were used for RNA isolation. For first instar brain samples, total RNA was isolated using Trizol (Invitrogen) following manufacturer's directions. Samples were DNAsed and reverse transcribed using oligo dT primers. The resulting cDNA was used to perform quantitative Real Time PCR with SYBR Green dye. For ventral ganglia isolated during second instar RNA was extracted and the sequences amplified as described in [49,50]. Hemocyte studies were carried out on pooled hemolymph from three third instar larvae per sample. RNA was extracted and amplified as for ventral ganglia. All qRT-PCR reactions were carried out in triplicate at three different template concentrations to ensure that we were within linear template range. Primer sequences are available upon request. *β-actin *expression was used as an internal control. Data were analyzed using the delta-delta calculation method to yield fold change compared to controls.

### Statistics

Determination of significance was accomplished by use of Student's t test or ANOVA, depending on the design of the study.

## List of Abbreviations

Bnl: Branchless

BrdU: 5-Bromodeoxyuridine

dpp: decapentaplegic

FGF: Fibroblast Growth Factor

GFP: Green Fluorescent Protein

Hh: Hedgehog

HSPG: Heparan Sulfate Proteoglycan

PDGF: Platelet Derived Growth Factor

PVR: PDGF- and VEGF-Receptor Related

qRT-PCR: quantitative Real Time PCR

SHH: Sonic Hedgehog

TGFβ: Transforming Growth Factors

TNb: Thoracic neuroblast

VEGF: Vascular Endothelial Growth Factor

wg: wingless

## Authors' contributions

JL carried out all of the TNb genetic and molecular analyses, localization of Trol-GFP in the larval brain, hemocyte counting and qRT-PCR in hemocytes. JL also oversaw the *trol *developmental/lethality phenotypic analyses.

PH carried out the *trol *developmental/lethality analyses and participated in the *trol *TNb studies.

AB oversaw the *trol *hemocyte counting studies.

MC participated in the *trol *hemocyte counting studies.

TP carried out the qRT-PCR analyses of first instar *trol *larval brains.

YP carried out the BrdU study of the *trol*^7 ^first instar neuroblast phenotype.

SD conceived of the studies, contributed to the experimental design and interpretation of all studies and wrote the manuscript.

All authors have read and approved the final manuscript.
